# Virulence profiling of *Cryptococcus gattii* isolates in China: insights from a multi-center study

**DOI:** 10.1128/spectrum.02443-23

**Published:** 2023-10-31

**Authors:** Xuelei Zang, Weixin Ke, Yemei Huang, Chen Yang, Jialin Song, Hengyu Deng, Meng Zhou, Qiqi Wang, Yangyu Zhou, Bin Dai, Jin Qian, Dingxia Shen, Linqi Wang, Xinying Xue

**Affiliations:** 1 Department of Respiratory and Critical Care, Emergency and Critical Care Medical Center, Beijing Shijitan Hospital, Capital Medical University, Beijing, China; 2 State Key Laboratory of Mycology, Institute of Microbiology, Chinese Academy of Sciences, Beijing, China; 3 Capital Medical University, Beijing, China; 4 Medical School of Chinese PLA, Beijing, China; 5 Medical Laboratory Center, The First Medical Centre, Chinese PLA General Hospital, Beijing, China; 6 Weifang Medical University, Weifang, China; 7 School of Medical Imaging, Binzhou Medical University, Yantai, China; 8 Department of Neurosurgery, Beijing Shijitan Hospital, Capital Medical University, Beijing, China; Centro de Investigaciones Biologicas CSIC, Madrid, Spain

**Keywords:** *Cryptococcus gattii*, molecular epidemiology, virulence, multi-center study

## Abstract

*Cryptococcus gattii* (*C. gattii*) is emerging as a life-threatening fungus worldwide. However, few data were available in China. In this study, 27 isolates and 32 patient information were collected from several hospitals in China, together with previously reported data (80 cases), to describe the geographic distribution of *C. gattii*. Molecular identification and genetic diversity analysis of the isolates were conducted using multilocus sequence typing, while the microbiological and virulence characteristics were explored through *in vitro* phenotypic tests and *in vivo* animal experiments. The findings revealed that patients infected with *C. gattii* were mainly immunocompetent males, with most showing symptoms of central nervous system involvement. Isolated strains were predominantly distributed in tropical and subtropical regions, with VGI genotype predominance. These strains exhibited marked genetic diversity, identifying 25 distinct sequence types (STs) among the 57 isolates (available data), including three novel STs (ST565, ST567, and ST568). *In vitro* assays unveiled significant differences between VGI and VGII in growth capacity at 39°C, capsule diameter, melanin production, UV resistance, and antioxidative capacity, with VGII displaying greater resilience. Integrating animal experiments and clinical prognosis showed that pathogenicity did not directly correlate with *in vitro* virulence phenotypes or molecular genotypes, underscoring virulence’s complexity. Furthermore, histopathological analysis suggested that lung tissue damage might primarily contribute to mouse mortality, particularly with more pathogenic strains causing extensive lung tissue damage. Our study has the potential to provide valuable data for a comprehensive understanding of the microbiological characteristics of *C. gattii* in China.

**IMPORTANCE:**

Our study indicates that the molecular typing of *Cryptococcus gattii* is unrelated to virulence. The integration of animal experiments and clinical prognosis demonstrated that pathogenicity did not exhibit a direct correlation with *in vitro* virulence phenotypes or molecular genotypes, emphasizing the intricate nature of virulence. In conclusion, our research holds the potential to provide valuable insights into understanding the microbiological attributes of *C. gattii* in China.

## INTRODUCTION

As a pathogenic fungus that could infect immunocompetent individuals, *Cryptococcus gattii* tends to invade through the respiratory tract causing pulmonary cryptococcosis and can also spread to other organs, especially the central nervous system (CNS), causing meningoencephalitis (1). In 1999, a noteworthy outbreak of *C. gattii* infection was initiated among both human and animal populations on Vancouver Island, Canada (2). This outbreak subsequently propagated to the Pacific Northwest region of the United States (3). Through the identification of seven distinct genetic loci (*CAP59*, *GPD1*, *LAC1*, *PLB1*, *SOD1*, *URA5*, and *IGS1*), *C. gattii* could be taxonomically categorized into four distinct molecular types, denoted as VGI, VGII, VGIII, and VGIV (4). The study confirmed that VGII was the main type of *C. gattii* causing outbreaks on Vancouver Island. Specifically, VGIIa emerged as the primary causative subtype, followed by VGIIb as the secondary causative subtype. Additionally, the VGIIc subtype incited the outbreaks observed in the Pacific Northwest region of the United States (5).

Significant geographic diversity exists between the different molecular types of *C. gattii*. Initially classified as an endemic molecular type, VGI was notably confined to Australia and select regions (6) and now dominates in Europe, Asia, and elsewhere (7). VGII, in contrast, is prominently distributed within the Americas, spanning the United States, Canada, and Brazil (7 – 10). Meanwhile, VGIII is also widely distributed in the Americas (7), such as Mexico and Colombia (11, 12), with few reports from other locations.

The pivotal microbiological and physiological attributes of *C. gattii*, instrumental in facilitating its sustenance within human hosts, are of paramount significance in the context of human infections. These attributes encompass the capability to thrive at 37°C, possession of a capsule, melanin synthesis, and antioxidant proficiency. Notably, these characteristics have garnered affirmation through *in vitro* investigations, thus demonstrating a correlation with virulence (13 – 17). Moreover, an association between virulence and molecular typing has been discerned. Evidently, the pathogenic VGIIa isolate that is accountable for the malady on Vancouver Island, Canada, exhibited heightened virulence in comparison to its VGIIb counterpart (18), This conclusion was reinforced by *in vivo* virulence assessments, which unequivocally affirmed the heightened pathogenicity of VGIIa relative to VGIIb (3, 19).

In this study, isolates and clinical data were collected from several hospitals in China. This comprehensive data set facilitated the description of both molecular typing and the geographic dispersion of *C. gattii*, further integrated with previously documented clinical profiles of afflicted patients. Additionally, our study encompassed *in vitro* phenotypic assays alongside *in vivo* mouse experiments, thereby affording an exploration of virulence disparities among clinical isolates. We aim to provide useful data for the prevention and management of *C. gattii*.

## MATERIALS AND METHODS

### Isolate and clinical data collection from patients

Isolates and clinical data of individuals afflicted with *C. gattii* infection were retrospectively acquired from multiple medical institutions within China. Elaborate clinical records were meticulously compiled, encompassing demographic attributes such as gender, age, and underlying health conditions. Moreover, a comprehensive follow-up evaluation of patient prognosis was meticulously conducted.

### Retrieval of previously reported *C. gattii* data in China

Clinical data, hitherto documented in antecedent studies, were meticulously sourced from prior publications. This encompassed meticulous recording of geographic prevalence and molecular classification attributes pertaining to *C. gattii*. To facilitate comprehensive analysis, we downloaded genomic information for reference strains, specifically R265, R272, WM175, WM179, and WM779, from the National Center for Biotechnology Information.

### DNA extraction

DNA extraction was performed similarly to the previous study (20). The cells were grounded in liquid nitrogen, and cetyl trimethyl ammonium bromide was added and then incubated in the water bath at 65°C for 30 minutes, followed by extraction with chloroform and isopropanol.

### Multilocus sequence typing (MLST) and phylogenetic analysis

Following the guidelines outlined by the International Society for Human and Animal Mycology consensus MLST scheme (4), we performed gene amplification and sequencing of seven distinctive genes: *CAP59*, *GPD1*, *LAC1*, *IGS1*, *PLB1*, *SOD1*, and *URA5*. Subsequently, the genotype of each strain, alongside the associated sequence type (ST), was ascertained through meticulous comparison with the database (http://mlst.mycologylab.org).

To infer the phylogenetic relationships of the clinical isolates in China, we combined molecular information from previously reported strains and performed a phylogenetic analysis. A maximum likelihood phylogenetic tree was constructed using MEGA7 software with 1,000 bootstrap replicates for each hypervariable marker. Finally, the phylogenetic tree was generated by iTOL.

### Phenotypic testing

Strains were incubated in yeast extract–peptone–dextrose (YPD) liquid medium at 30°C for 16 hours, followed by centrifugation and two washes with phosphate-buffered saline. The strains were uniformly adjusted to 5 × 10^7^ cells/mL and then gradient diluted to 10^7^ cells/mL, 2 × 10^6^ cells/mL, 4 × 10^5^ cells/mL, 8 × 10^4^ cells/mL, and 1.6 × 10^4^ cells/mL.

The cells (3 µL from each concentration) were respectively plated onto YPD agar medium to assess the growth at different temperatures (30°C, 37°C, and 39°C). To analyze stress response phenotypes, cells (3 µL from each concentration) were, respectively, plated onto YPD agar medium containing different pressure conditions to observe the growth at 30°C. The pressure conditions were as follows: osmotic (2 M sorbitol) and cation/salt stresses (1.5 M NaCl and 1.5 M KCl); oxidative stress (0.03% H_2_O_2_ and 3 mM diamide); ionizing radiation (253.7 nm, 1 minute); wall-destabilizing stress (2% Congo red) (21). Melanin production was induced using a minimal medium containing L-3,4-dihydroxyphenylalanine (0.1 g/L). Cells were incubated at 30°C for 3 days and then photographed (16). Capsule formation was induced by incubating cells at a uniform concentration of 1 × 10^5^ cells/mL and culturing them at 37°C. A mixture of 4 µL of cell suspension and 4 µL of ink was prepared on a microscope slide, and images of cell morphology were captured using a Carl Zeiss microscope equipped with software (Zen 2011). Twenty normal cells were selected to measure the total diameter (including capsule) (TD) and yeast cell diameter (YD). Capsule thickness (CT) was calculated as $\frac{1}{2}(TD-YD)$ .

### Mouse experiments

To assess the variance in virulence between VGI and VGII, all isolates and reference strains (R265, R272, and WM276) underwent survival testing. To establish the murine inhalation model for conducting animal experiments, the specific procedures are as follows: Cell concentrations were adjusted to 2 × 10^6^ cells/mL. Experiments were carried out using female C57BL/6 mice aged 8–10 weeks, with 10 mice utilized to evaluate virulence for each strain. After the mice were anesthetized with 1.25% of avertin, 50 µL (2 × 10^6^ cells/mL) was slowly dropped into the nasal cavity of each mouse. Mice were observed every day for 90 days, and the deaths were recorded.

### Pathologic analysis

Three mice per group were used for pathological studies, and the mice infected with isolates were euthanized at 14 days. Lung tissue from the mice was utilized for histopathological examination.

Hematoxylin and eosin (HE) staining of lung tissues was performed as previously described. Briefly, the entire lung of mice infected with *C. gattii* was dissected, fixed with 4% paraformaldehyde, and embedded with paraffin. A maximum cross-section was selected and stained. Similarly, lung tissues were stained with Periodic acid–Schiff (PAS) and also sealed with dehydration.

The complete sections were scanned for analysis using Pannoramic Midi: 3D/Histech. Visualization of the stained section was accomplished using CaseViewer, version 2.4.2.8.

### Statistical analysis

All data were analyzed by SPSS 19.0 software (IBM Corporation, USA). Descriptive statistical methods were used to summarize the clinical characteristics of the patients. Continuous data were expressed as mean ± standard deviation. The phenotypic characteristics of the strains were scored based on the results of phenotypic tests (strongly resistant or enhanced: 3; moderately resistant or enhanced: 2; weakly resistant or enhanced: 1; WM276-like phenotype: 0; weakly sensitive or defective: −1; moderately sensitive or defective: −2; and strongly sensitive or defective: −3). Phenotypic traits between the VGI and VGII groups were analyzed by Mann-Whitney. Survival analysis was performed by log-rank test. Plots were generated using GraphPad Prism 8.0 version. *P*-value < 0.05 was considered a significant difference.

## RESULTS

### Isolates and patients

In this study, a total of 32 isolates of *C. gattii* were collected from several hospitals in China, including patient information, and stored in glycerol broth at −80°C. Of these isolates, 27 were viable and included in this study. Furthermore, our endeavors encompassed the meticulous collection of data pertaining to an additional 80 strains of *C. gattii*, hitherto documented within China. This cumulative data set culminated in the aggregation of information relating to a total of 112 isolates. This amalgamated compendium encompassed 32 strains originating from our study and an additional 80 strains sourced from established databases. However, it is pertinent to acknowledge that, due to lacunae in the available data, information on STs was discernible for only a subset of 57 of these strains. A cohort comprising 32 individuals, all afflicted by *C. gattii* infection and possessing clinical data of relatively comprehensive nature, was judiciously recruited. Within this assemblage, a notable 27 individuals (accounting for 84.4%) were of male gender, while the remaining five individuals (constituting 15.6%) represented the female counterpart. The mean age, calculated at 44 years, exhibited a range spanning from 21 to 83 years. Furthermore, among the encompassed patient demographic, a striking 29 cases (90.6%) exhibited the absence of underlying pathologies and evinced normal immune function. Predominantly, a constellation of CNS manifestations, encompassing symptoms such as fever, headache, and vomiting, was notably prevalent. This assemblage of symptoms was conspicuously discernible in a substantial cohort comprising 29 patients (90.6%). The strains primarily exhibited isolation from the cerebrospinal fluid (CSF) domain, an observation reinforced by the fact that solely two strains were isolated from alternate anatomical locales. Specifically, one strain was derived from sputum, while the other originated from lung tissue. Furthermore, a favorable prognosis was distinctly discernible among 28 patients (reflecting a notable 87.5% of the cohort), whereas a somber outcome was witnessed in only four individuals who succumbed to the ailment (Table 1).

**TABLE 1:** Clinical features of patients infected with *C. gattii*

Characteristic	Total (*n* = 32)	Percentage (%)
Age (years)	44.3 ± 13.6	
Age range (years)	20–81	
Gender (male)	27	84.3
Immunocompetent	29	90.6
Underlying disease	3	9.4
Strain source		
CSF	29	90.6
Sputum	1	3.1
Lung tissue	2	6.3
Prognosis		
Improve	28	87.5
Death	4	12.5
Symptoms		
CNS symptoms (including fever/headache/vomiting)	29	90.6
Only cough	3	9.4

### Geographical distribution

**Fig 1:**
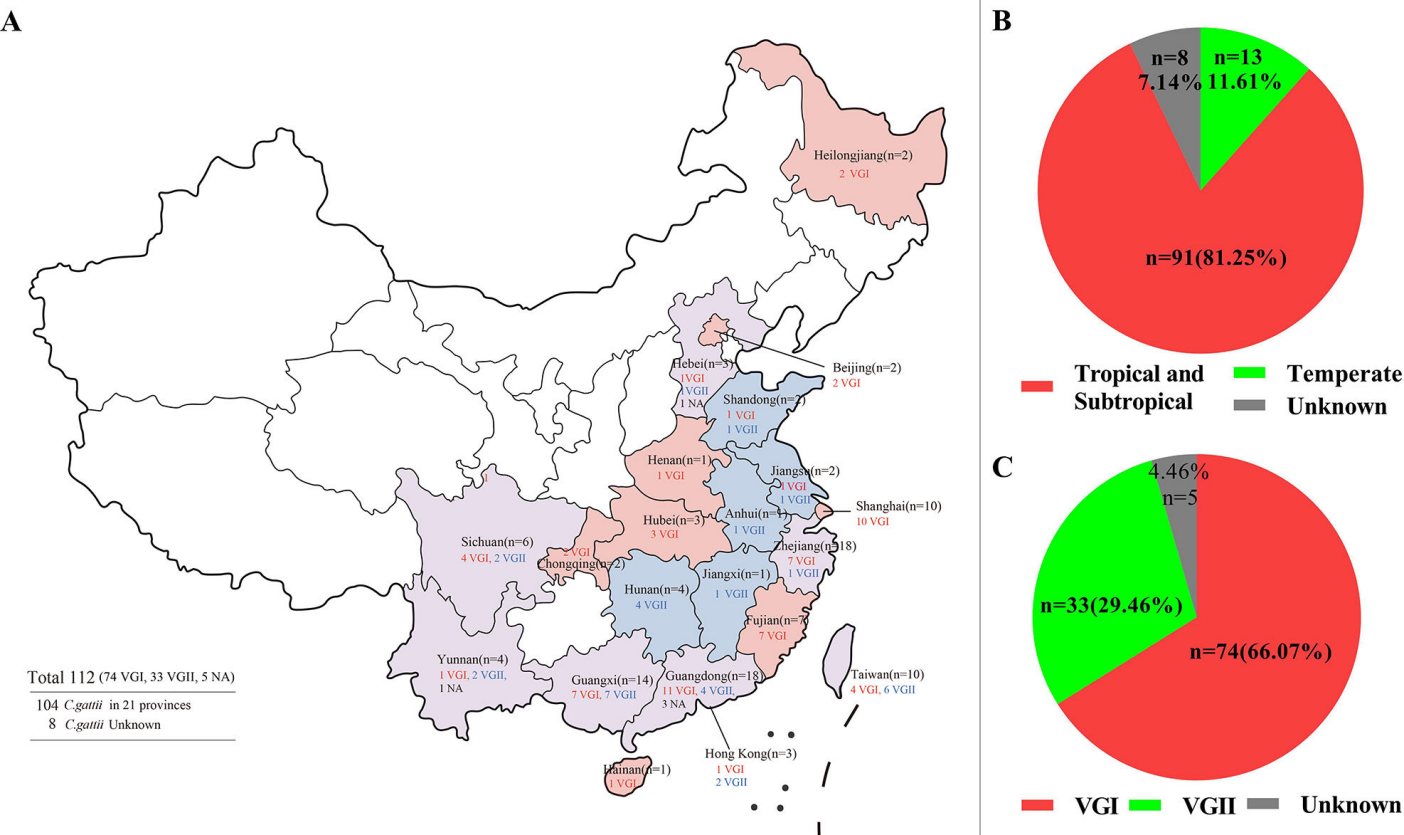
Molecular epidemiological characteristics of *C. gattii* from China involved in this study. (**A**) Map of molecular typing and geographical distribution of *C. gattii* from China by province. Map created using Photoshop AI. (**B**) Climatic features of the geographical distribution of *C. gattii* from China. (**C**) The percentage distribution of different molecular typing of *C. gattii* from China.

In pursuit of a comprehensive and precise depiction of the geographical distribution, we meticulously scrutinized the entirety of the available strain data. A total of 112 instances of *C. gattii* were documented, spanning across no fewer than 21 provincial-level administrative regions within China. Predominantly clustered within the southeastern coastal regions characterized by subtropical climates, the majority of isolates were discernibly congregated. Intriguingly, a minor subset of isolates had likewise commenced emerging within the northeastern locales marked by temperate climatic conditions. A discernible inverse correlation was evident between case frequency and latitude, whereby the incidence of cases diminished with the increase in latitude. Additionally, our data set revealed that the VGI molecular type prevails, constituting a significant 66.07% of the documented cases (Fig. 1).

### Genetic variability

**Fig 2:**
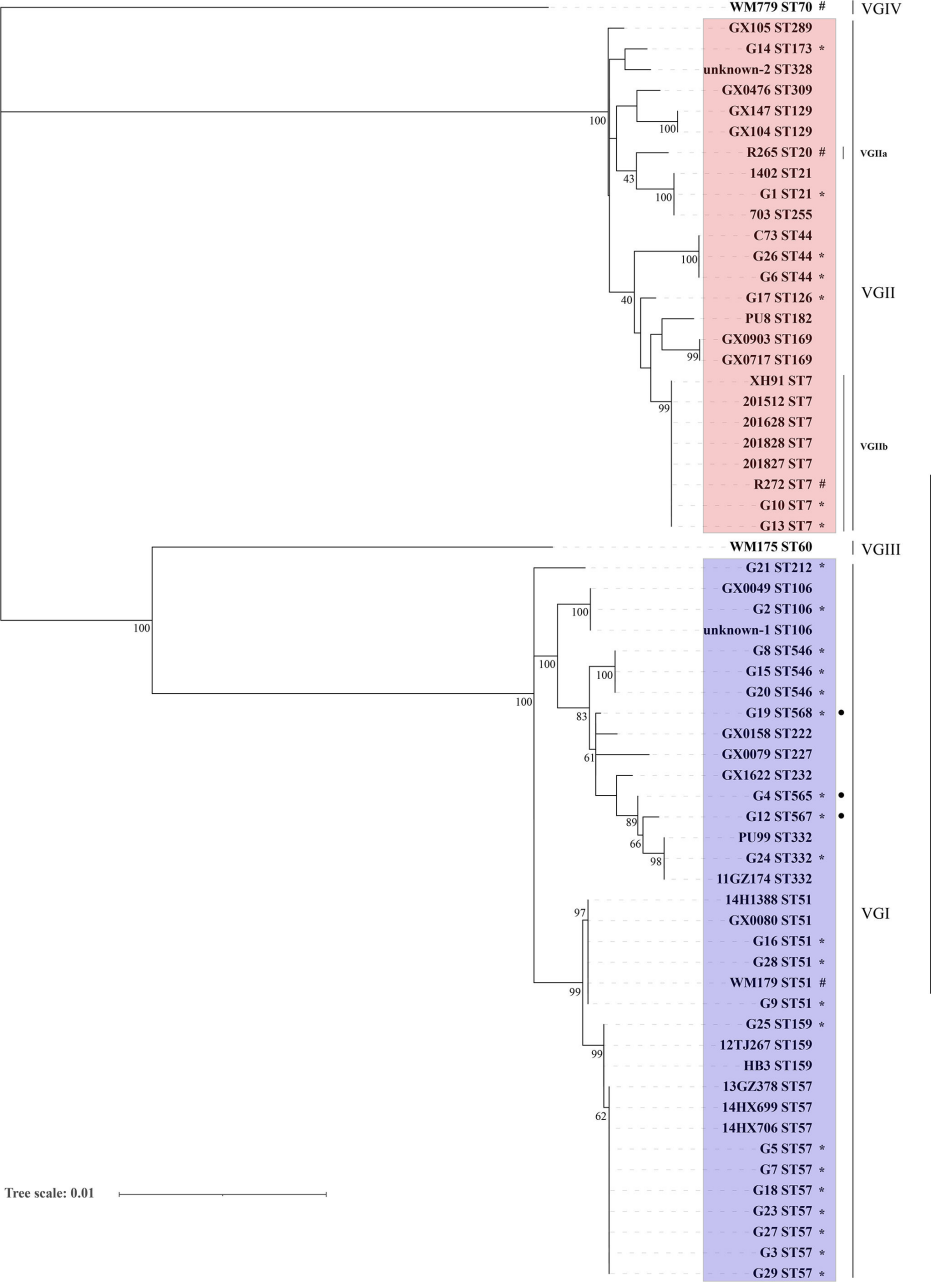
Phylogram based on MLST analysis showing the genetic relationships of *C. gattii* isolates from China involved in this study, presenting 25 STs in 57 isolates. **•**Novel sequence types (STs) identified in this study. *The isolates collected in the present study. #The reference strains collected in this study.

A total of 27 viable isolates, gathered for this study, underwent meticulous MLST analysis. This assemblage comprised 7 strains of VGII and 20 strains of VGI. The results revealed a pronounced genetic diversity exhibited by the clinical strains, culminating in the identification of 15 STs. Most notably, ST57 emerged as the predominant variant, encompassing seven distinct strains. Noteworthy to mention is the discovery that G10 and G13 shared an identity with ST7 (VGIIb), corresponding to the minor clonal strain identified during the Vancouver Island outbreak. Importantly, the absence of ST20 (VGIIa), the principal clonal strain associated with the Vancouver Island outbreak, within the Chinese data set remains evident. Furthermore, the investigation yielded the identification of three previously unreported STs: ST565 (G4), ST567 (G12), and ST568 (G19). In an endeavor to deepen the scrutiny into the molecular evolution of domestic strains, a phylogenetic tree was constructed based on the existing molecular information encompassing 57 instances (Table S1). The outcomes of this endeavor underscored the inherent genetic heterogeneity within the domestic strains, which resulted in the detection of 25 unique ST. Prominent was ST57, which accounted for 10 distinct strains, followed by ST7 with seven strains. Notably, ST20 was absent within the Chinese data set. (Fig. 2).

### Phenotypic characteristics of strains

**Fig 3:**
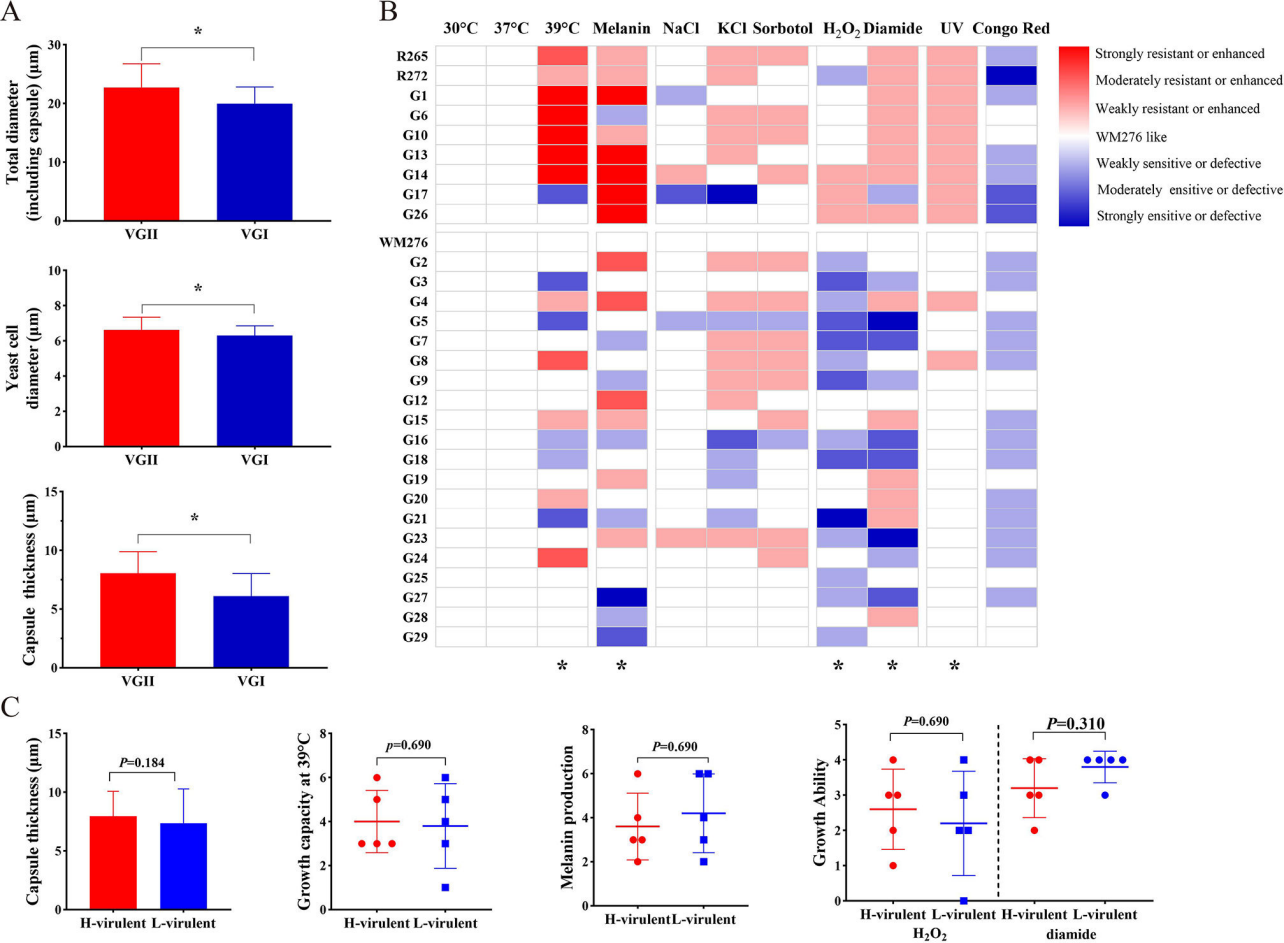
Results of *in vitro* phenotyping experiments for all strains in the present study. (**A**) The total diameter, cell diameter, and capsule diameter were measured for 20 cells of *C. gattii*. Error bars indicating SD. (**B**) Heat map based on phenotype scores, with red and blue in the heat map representing strongly resistant or enhanced and weakly resistant or defective, respectively. Phenotypic strengths are distinguished by the gradient of red or blue. (**C**) Five highly virulent strains (R265, WM276, G14, G25, and G9) and less virulent strains (R272, G8, G21, G26, and G13) were selected for grouping, with the aim of analyzing the differences in *in vitro* virulence phenotypes between the two groups. Error bars indicating SD.

Growth disparities among all isolates were observed under various stress resistance conditions, and we assigned scores based on their growth behavior. Our results indicated variations between VGI and VGII strains in terms of growth capacity at 39°C, melanin production, resistance to oxidative stress, and UV resistance. In contrast, notable differences were not detected in cell wall and osmotic stress resistance. Clinical strains of VGI presented an average capsule thickness of 6.83 µm, which was significantly thinner than that of VGII (8.05 µm) (Fig. 3A and B).

### Virulence study of isolates

**Fig 4:**
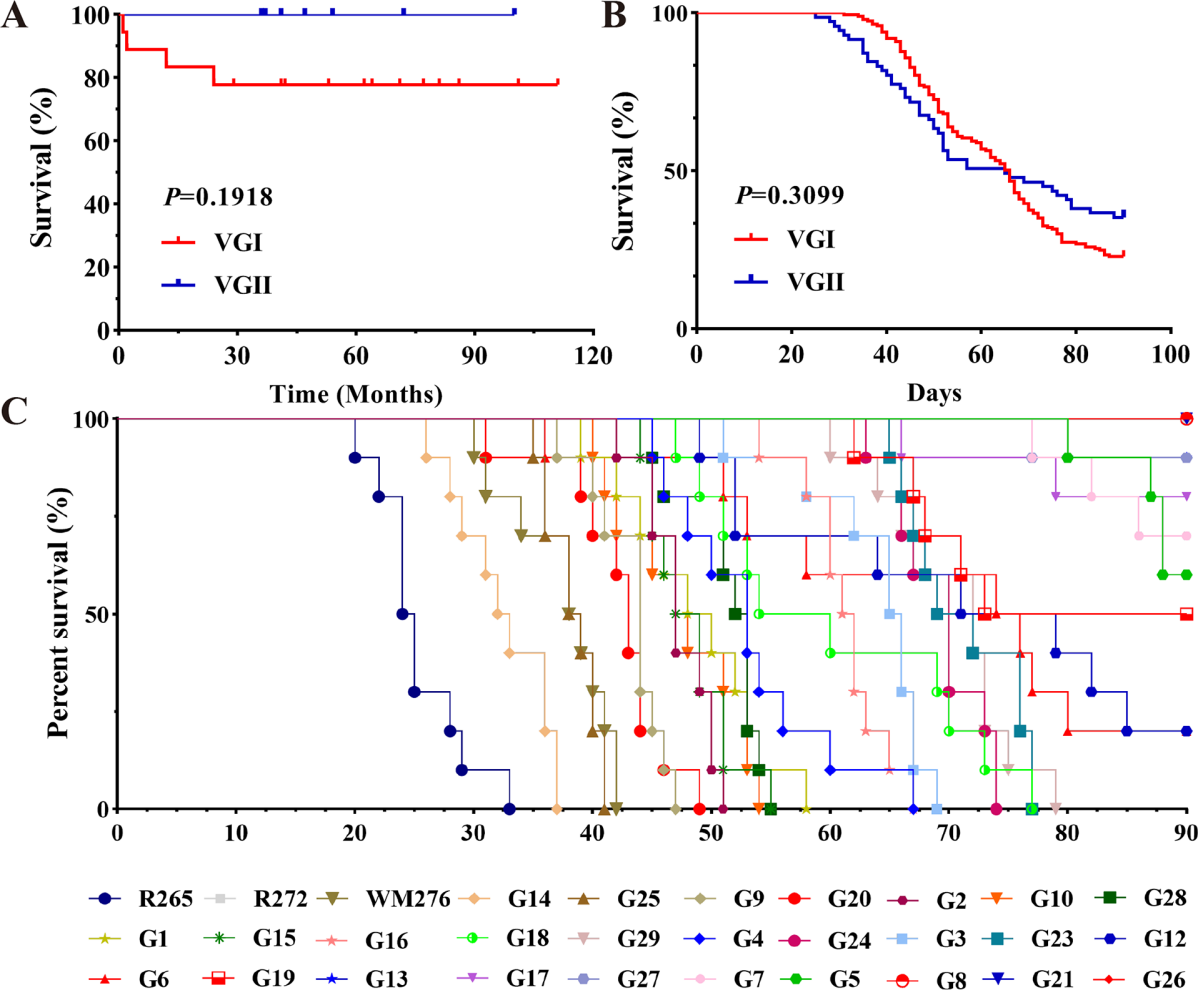
No significant differences in virulence were observed between VGI and VGII in animal studies and clinical prognosis. (**A**) Survival curves for patients infected with VGI and VGII *C. gattii* based on clinical follow-up information. (**B**) Survival curves of mice infected with VGI and VGII *C. gattii.* (**C**) The survival curves of individual mice following *C. gattii* infection are depicted, with distinct colors denoting subgroups of infecting strains. *P < 0.05.

All patients were followed for survival, and the results showed no significant difference in the overall survival between patients infected with VGI and VGII (Fig. 4A).

Subsequently, an *in vivo* assay was conducted to assess the virulence of isolates using a murine inhalation model. Survival curves demonstrated distinct pathogenicity among the strains in mice. Within the reference strains, mice infected with R265 exhibited the shortest survival period, reflecting its heightened virulence, followed by WM276; R272 showcased the least virulence. Among clinical isolates, G14-induced infections led to the shortest survival time, thereby designating G14 as the most virulent strain, trailed by G25 and G9. Notably, G8, G21, and G26 failed to induce mice mortality, establishing them as the least virulent isolates. Overall, despite VGII-infected mice dying at an earlier time point than their VGI counterparts, statistical analysis revealed no significant variance in virulence between the two molecular types (Fig. 4B). In contrast, we noted variations in virulence even among strains of the same molecular subtype. For example, within the VGIIb, both G10 and G13 displayed greater virulence than R272 (Fig. 4C).

To evaluate the relationship between phenotypes and virulence, we selected, based on survival curves, five highly virulent strains (R265, WM276, G14, G25, and G9) and five low-virulence strains (R272, G8, G21, G26, and G13) for analysis. The results demonstrate that commonly considered virulence factors, including high-temperature tolerance, antioxidative capacity, melanin production, and capsule size, were not correlated with virulence levels (Fig. 3C).

### Pathological characteristics of lung tissues infected with isolates

**Fig 5:**
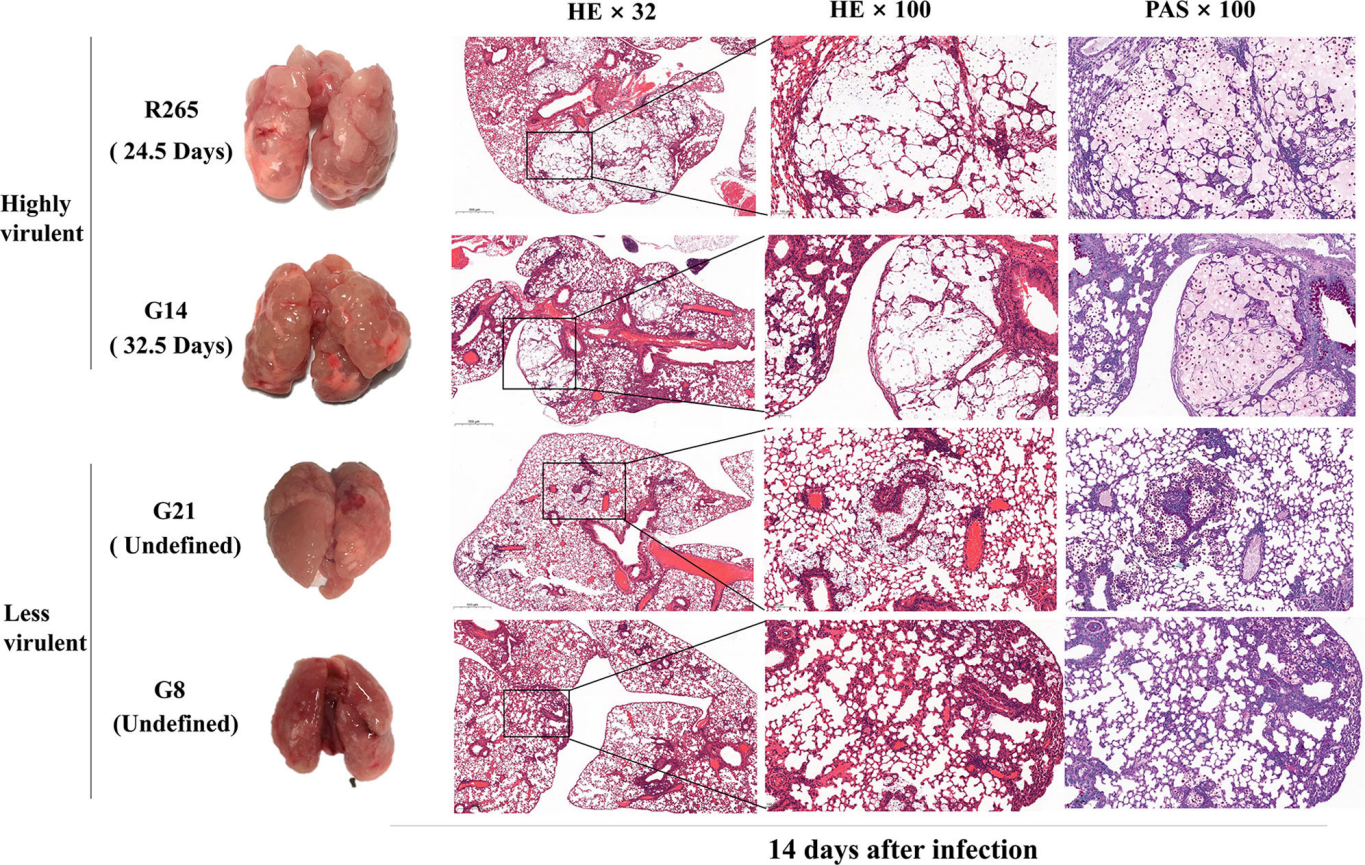
Pathological differences in the lung tissue of mice infected with highly virulent and less virulent strains. After 14 days post-infection, the staining results showed greater damage to lung tissue from the highly virulent strains. The images were captured by microscope (×32, ×100), respectively. The numbers in parentheses represent the median time to death.

HE staining and PAS staining were conducted on lung tissues from mice at 14 days post-infection. Based on survival curves, two strains with high virulence (R265 and G14) and two strains with lower virulence (R272 and G26) were chosen for subsequent pathological investigations. Mice infected with the highly virulent strains displayed edema and irregular bulges on the lung surface. Concurrently, the alveolar septa exhibited pronounced thickening and structural alterations, accompanied by distinct pulmonary enlargement. Furthermore, a substantial number of fungal cells were dispersed throughout the alveolar cavity. In contrast, lung tissues of mice infected with less virulent strains exhibited relatively normal surfaces, while cells clustered within the alveolar cavities (Fig. 5). These findings suggest that highly virulent strains inflict greater damage on lung tissue compared to their less virulent counterparts. We conclude that the primary contributor to mortality in the mice was the destruction of lung tissue.

## DISCUSSION

Our findings align with prior research indicating heightened susceptibility among males and immunocompetent individuals to *C. gattii* infection (1, 22). Unlike patients in the Vancouver Island outbreak region, who primarily exhibited pneumonia (23, 24), the majority of our patients presented with CNS symptoms. On the whole, most patients achieved a satisfactory recovery following diagnosis and treatment. Notably, patients displaying CNS symptoms may be more readily identified, facilitating timely access to treatment.

The prevalence of *C. gattii* is primarily observed within tropical and subtropical regions, where the combination of elevated rainfall and temperatures in humid environments provides conducive conditions for the proliferation of isolates (25). Furthermore, certain isolates have been identified in temperate regions, extending their presence northward to the Heilongjiang province. This observation underscores the strains' remarkable ecological adaptability, enabling their survival across a spectrum of environments, encompassing dry, humid, warm, and even cold conditions (19, 26).

This study suggests that the predominant molecular type of *C. gattii* in China is the VGI genotype, accompanied by considerable genetic variability among isolates. This diversity is evident with the identification of 25 STs within 57 isolates, which encompasses three novel STs. Of particular concern is the prevalence of ST7 (a minor clonal subgroup of the Vancouver Island outbreak strain) in China, which is also widely distributed across other regions including Canada, Brazil, and Asia (2, 27). It has been suggested that Australia served as the source of the Vancouver Island outbreak (4), along with the proposition that clonal strains of this genotype disseminated globally via the transport of *Eucalyptus camaldulensis* (28), and it has been speculated that *C. gattii* in China might also have been spread through *E. camaldulensis* (29, 30). However, unfortunately, no environmental strains have been obtained, and further studies are needed to find the true route of transmission. Despite the absence of ST20 (the predominant clonal subgroup of the Vancouver Island outbreak strain) within China, phylogenetic analysis of the evolutionary tree revealed a close relationship between the clinical isolate G1 and the profoundly virulent R265 strain responsible for the outbreak’s inception.

The capsule thickness of VGII significantly surpassed that of the VGI type, aligning with previous research findings. Moreover, notable dissimilarities emerged among the assessed isolates, encompassing variations in growth ability at 39°C, melanin production capacity, and UV resistance. Notably, VGII exhibited substantially greater strength compared to VGI. Despite the perception of these phenotypic traits as potential correlates of virulence, no discernible distinctions in virulence between VGI and VGII have been ascertained through animal models and clinical prognosis. Furthermore, intriguingly, G10, G13, and R272 all belong to the VGIIb subtype; however, the virulence of G10 and G13 strains eclipses that of R272. This outcome indicates that strains of identical molecular types can manifest differential virulence, even within the confines of a single subtype. Similarly, in our *in vitro* experiments, we found that variations in strain resistance to elevated temperatures, capsule thickness, antioxidative capacity, and melanin production were not directly correlated with the mortality rates of infected mice. This underscores the complexity of *C. gattii* virulence, suggesting that a single phenotypic trait cannot fully capture differences in virulence between strains. Several studies have suggested that virulence is determined by the regulated expression of various factors as well as complex genetic traits (15, 31, 32). Our data support this perspective, further validating the idea that virulence in *Cryptococcus* is shaped by a multifaceted interplay of factors.

The examination of infected mice revealed histological changes within their lungs. These alterations likely represent the primary contributors to the demise of the mice. Furthermore, it was observed that the virulent isolates exhibited heightened destructiveness to lung tissue.

It is important to acknowledge the limitations of this study, as the relatively restricted number of available isolates might hinder a comprehensive elucidation of the distribution and virulence characteristics within *C. gattii* from China. In conclusion, despite these limitations, our study imparts valuable insights for strategies pertaining to the prevention and control of *C. gattii*.

## AFTER EPUB

*[This article was published on 31 October 2023 with errors in the affiliation numbering in the byline. The errors were corrected in the current version, posted on 7 November 2023.]*

## SUPPLEMENTAL MATERIAL

Table S1 spectrum.02443-23-s0001.docx Information on *C. gattii* collected from PubMed and Chinese academic databases.

ASM does not own the copyrights to Supplemental Material that may be linked to, or accessed through, an article. The authors have granted ASM a non-exclusive, world-wide license to publish the Supplemental Material files. Please contact the corresponding author directly for reuse.
