# Supplementary material for: Virulence profiling of Cryptococcus gattii isolates in China: insights from a multi-center study
Source: Microbiol Spectr. 2023 Oct 31;11(6):e02443-23. doi: 10.1128/spectrum.02443-23 (PMC10714995; doi:10.1128/spectrum.02443-23)
Supplement: Table S1 — Information on C. gattii collected from PubMed and Chinese academic databases. [file spectrum.02443-23-s0001.docx]

Table S1: The information on *C.gattii* collected from PubMed and Chinese academic databases

| Strain ID | Province | Isolation Date | Isolation Location | MLST Type | Genotyping | Reference |
| --- | --- | --- | --- | --- | --- | --- |
| 1402 | Hong Kong | 2001 | CSF | 21 | VGII | [1] |
| 703 | Hong Kong | 2003 | CSF | 255 | VGII | [1] |
| 337 | Hong Kong | 1998 | CSF | - | VGI | [1] |
| XH91 | Guangdong | 2005 | CSF | 7 | VGII | [2] |
| XH23 | Eastern China | 2000 | CSF | - | VGI | [2] |
| XH24 | Eastern China | 2000 | CSF | - | VGI | [2] |
| XH3 | Eastern China | 1981 | CSF | - | VGI | [2] |
| XH31 | Eastern China | 2001 | CSF | - | VGI | [2] |
| XH32 | Eastern China | 2001 | Sputum | - | VGI | [2] |
| XH4 | Eastern China | 1993 | CSF | - | VGI | [2] |
| XH5 | Eastern China | 1993 | CSF | - | VGI | [2] |
| XH72 | Eastern China | 2004 | CSF | - | VGI | [2] |
| PU99 | Guangdong | 2011 | CSF | 332 | VGI | [3] |
| PU8 | Hebei | 2008 | CSF | 182 | VGII | [3] |
| C73 | Guangxi |  | CSF | 44 | VGII | [4] |
| HB3 | Hubei |  | CSF | 159 | VGI | [4] |
| 13GZ378 | Guangdong |  |  | 57 | VGI | [5] |
| 11GZ174 | Guangdong |  |  | 332 | VGI | [5] |
| 14H1388 | Heilongjiang |  |  | 51 | VGI | [5] |
| 12TJ267 | Hubei |  |  | 159 | VGI | [5] |
| 12HX537 | Sichuan |  |  | - | VGII | [5] |
| 14HX699 | Guangdong |  |  | 57 | VGI | [5] |
| 14HX706 | Guangdong |  |  | 57 | VGI | [5] |
| NA | Zhejiang |  |  | 328 | VGII | [6] |
| BJ001 | Beijing |  | CSF | - | VGI | [7] |
| GX0080 | Guangxi |  | CSF | 51 | VGI | [8] |
| GX0049 | Guangxi |  | CSF | 106 | VGI | [8] |
| GX104 | Guangxi |  | CSF | 129 | VGII | [8] |
| GX147 | Guangxi |  | CSF | 129 | VGII | [8] |
| GX0717 | Guangxi |  | lung tissue | 169 | VGII | [8] |
| GX0903 | Guangxi |  | CSF | 169 | VGII | [8] |
| GX0158 | Guangxi |  | CSF | 222 | VGI | [8] |
| GX0079 | Guangxi |  | CSF | 227 | VGI | [8] |
| GX1622 | Guangxi |  | CSF | 232 | VGI | [8] |
| GX105 | Guangxi |  | CSF | 289 | VGII | [8] |
| GX0476 | Guangxi |  | CSF | 309 | VGII | [8] |
| 201512 | Hunan |  | CSF | 7 | VGII | [9] |
| 201628 | Hunan |  | CSF | 7 | VGII | [9] |
| 201827 | Hunan |  | CSF | 7 | VGII | [9] |
| 201828 | Hunan |  | CSF | 7 | VGII | [9] |
| CHC-15 | Zhejiang | 2004 | CSF |  | VGI | [10] |
| CHC-16 | Zhejiang | 2004 | CSF |  | VGI | [10] |
| CHC-24 | Guangdong | 2006 | CSF |  | VGI | [10] |
| CHC-40 | Zhejiang | 2003 | CSF |  | VGI | [10] |
| CHC-154 | Shandong | 1995 | CSF |  | VGI | [10] |
| CHC-201 | Zhejiang | 1996 | CSF |  | VGI | [10] |
| CHC-203 | Jiangsu | 1994 | CSF |  | VGI | [10] |
| CHC-209 | Shanghai | 2005 | CSF |  | VGI | [10] |
| isolate9 | Zhejiang |  |  |  | VGI | [10] |
| SHCZ73 | Guangdong | 2005 | CSF |  | VGII | [11] |
| SHCZ20 | Shanghai | 1999 | CSF |  | VGI | [11] |
| SHCZ23 | Shanghai | 2000 | CSF |  | VGI | [11] |
| SHCZ24 | Shanghai | 2000 | CSF |  | VGI | [11] |
| SHCZ40 | Shanghai | 2002 | CSF |  | VGI | [11] |
| SHCZ5 | Shanghai | 1994 | CSF |  | VGI | [11] |
| SHCZ55 | Shanghai | 2004 | CSF |  | VGI | [11] |
| SHCZ6 | Shanghai | 1995 | CSF |  | VGI | [11] |
| SHCZ76 | Shanghai | 2006 | CSF |  | VGI | [11] |
| SHCZ82 | Shanghai | 2006 | CSF |  | VGI | [11] |
| SHCZ31 | Zhejiang | 2001 | CSF |  | VGI | [11] |
| SHCZ60 | Zhejiang | 2004 | CSF |  | VGI | [11] |
| - | Taiwan |  |  |  | VGI | [12] |
| T315 | Taiwan |  | CSF |  | VGII | [13] |
| T305 | Taiwan |  | CSF |  | VGII | [13] |
| T124 | Taiwan |  | CSF |  | VGI | [13] |
| T054 | Taiwan |  | CSF |  | VGI | [13] |
| T169 | Taiwan |  | CSF |  | VGII | [13] |
| T317 | Taiwan |  | CSF |  | VGI | [13] |
| T107 | Taiwan |  | CSF |  | VGII | [13] |
| T174 | Taiwan |  | CSF |  | VGII | [13] |
| T234 (T228) | Taiwan |  | Pleural effusion, (CSF) |  | VGII | [13] |
| isolate | Guangdong |  |  | 106 | VGI | [14] |
| - | Fujian |  |  |  | VGI | [15] |
| - | Fujian |  |  |  | VGI | [15] |
| - | Fujian |  |  |  | VGI | [15] |
| - | Fujian |  |  |  | VGI | [15] |
| - | Guangdong |  |  |  | VGI | [16] |
| - | Guangdong |  |  |  | VGII | [16] |
| - | Guangdong |  |  |  | VGII | [16] |
| GX11 | Guangxi |  |  |  | VGI | [17] |

A total of 80 reported strain information had been collected (some data points missing), including STs information for 30 strains.

Reference

[1] Lui G,Lee N,Ip M, et al.Cryptococcosis in apparently immunocompetent patients.QJM.2006.99(3):143-151.

[2] Feng X,Yao Z,Ren D, et al.Genotype and mating type analysis of Cryptococcus neoformans and Cryptococcus gattii isolates from China that mainly originated from non-HIV-infected patients.FEMS Yeast Res.2008.8(6):930-938.

[3] Dou HT,Xu YC,Wang HZ, et al.Molecular epidemiology of Cryptococcus neoformans and Cryptococcus gattii in China between 2007 and 2013 using multilocus sequence typing and the DiversiLab system. Eur J Clin Microbiol Infect Dis.2015.34(4):753-762.

[4] Wu SY,Lei Y,Kang M, et al.Molecular characterisation of clinical Cryptococcus neoformans and Cryptococcus gattii isolates from Sichuan province, China.Mycoses.2015.58(5):280-287.

[5] Fan X,Xiao M,Chen S, et al.Predominance of Cryptococcus neoformans var. grubii multilocus sequence type 5 and emergence of isolates with non-wild-type minimum inhibitory concentrations to fluconazole: a multi-centre study in China.Clin Microbiol Infect.2016.22(10):887.e1-887.e9.

[6] Fang LF,Zhang PP,Wang J, et al.Clinical and microbiological characteristics of cryptococcosis at an university hospital in China from 2013 to 2017.Braz J Infect Dis.2020,24(1):7-12.

[7] Chen M,Hong N,Hu S, et al.Molecular identification of Cryptococcus gattii from cerebrospinal fluid using single-cell sequencing: A case study.J Infect.2020.81(4):634-638.

[8] Huang C,Tsui CKM,Chen M, et al.Emerging Cryptococcus gattii species complex infections in Guangxi, southern China. PLoS Negl Trop Dis.2020.14(8):e0008493.

[9] Li Y,Zou M,Yin J, et al.Microbiological, Epidemiological, and Clinical Characteristics of Patients With Cryptococcal Meningitis at a Tertiary Hospital in China: A 6-Year Retrospective Analysis.Front Microbiol.2020.11:1837.

[10] Chen J,Varma A,Diaz MR, et al.Cryptococcus neoformans strains and infection in apparently immunocompetent patients, China.Emerg Infect Dis.2008.14(5):755-762.

[11] Chen, M., Li, XR., Wu, SX. et al. Molecular epidemiology of Cryptococcus neoformans species complex isolates from HIV-positive and HIV-negative patients in southeast China. Front. Med. China. 2010.4, 117–126.

[12] Liaw SJ,Wu HC,Hsueh PR. Microbiological characteristics of clinical isolates of Cryptococcus neoformans in Taiwan: serotypes, mating types, molecular types, virulence factors, and antifungal susceptibility.Clin Microbiol Infect.2010.16(6):696-703.

[13] Tseng HK,Liu CP,Ho MW, et al.Microbiological, epidemiological, and clinical characteristics and outcomes of patients with cryptococcosis in Taiwan, 1997-2010.PLoS One.2013.8(4):e61921.

[14] PH Guo, H Huang, XL Liu, et al. Multilocus sequence typing and clinical characteristics of cryptococcus neoformans in Guangdong province. Chin J Nosocomiol.2016.26(22):5072-5075. (in chinese)

[15] JT Zhu. A Study of Genotype and in Vitro Drug Sensitivity of 51 Cryptococcus Neoformans in Fujian Area. Master thesis. Fujian Medical University.2015.

[16] Xh Liang, JS Wu, XB Feng, et al. Molecular epidemiological study of Cryptococcus neoformans and Cryptococcus gattii in Shenzhen. Chinese Journal of Epidemiology. 2014.32(04):204-208. (in chinese)

[17] FN Bing, Y Wu, SB Yu, et al. Study on genotype and virulence of Cryptococcus neoformans and Cryptococcus gattii clinical isolates in Guigang,Guangxi Zhuang Autonomous Region. Chinese Journal of Epidemiology.2015.36(05):491-495. (in chinese)
